# Supplementary material for: Composition of Culturable Microorganisms in Dusts Collected from Sport Facilities in Finland during the COVID-19 Pandemic
Source: Pathogens. 2023 Feb 16;12(2):339. doi: 10.3390/pathogens12020339 (PMC9963892; doi:10.3390/pathogens12020339)
Supplement: Supplementary file 1 [file pathogens-12-00339-s001.zip › pathogens-2121542-supplementary.pdf]

**Table S1.** Morphology of major genera of fungal strains isolated from settled indoor dusts collected in sport facilities in Finland. Pathogenic potential was estimated based on the ability to grow at 37°C on Tryptic Soy Agar (TSA), pH 7.2, compared to growth on Malt Extract Agar (MEA), pH 5.5. The strains represent 67% of the total number of colonies.

| | **Samples positive on genus and (MT)** | | **Growth at 37°C*** | | **Conidia** | **Morphology** | |
|---|---|---|---|---|---|---|---|
| Genus/section | Nr. of isolates | Dusts samples positive | TSA | MEA | Size µm | Microscope | Colony on MEA |
| | | | | *Aspergillus* | | | |
| *Aspergillus* section *Nidulantes* | 5 | SF1 RD2 | - | - | 2—3 | 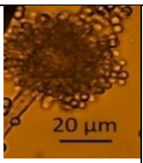 | 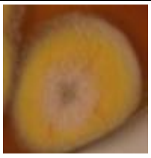 |
| *Aspergillus* section *Flavi* | 2 | SF1 SF2b | (+) | + | 4—5 | 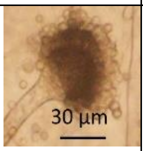 | 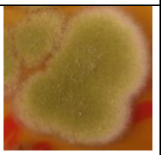 |
| *Aspergillus* section *Nigri* | 36 | Sf3a SF3b SF4 SF5 SF6 RD1 | + | + | 3—4 | 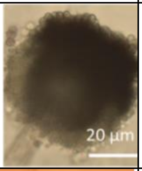 | 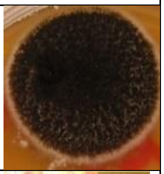 |
| *Aspergillus* section *Fumigati* | 10 | SF3a SF4 SF5 SF6 RD1 | + | + | 2—3 | 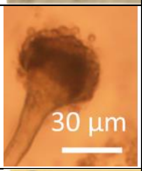 | 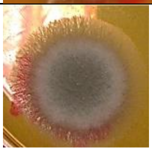 |
| *Aspergillus* section *Flavi* | 7 | SF4 SF5 SF6 SF7 | + | + | 4—5 | 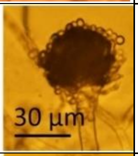 | 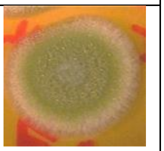 |
| *Aspergillus* section *Flavi* | 10 | SF3a SF3b SF4 SF5 SF6 | + | + | 4—6 | 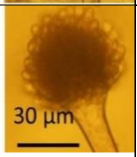 | 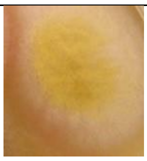 |
| *Aspergillus* section *Circumdati* | 1 | RD1 | - | - | 3—4 | 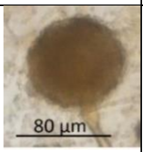 | 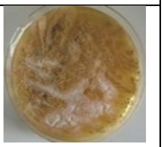 |
| | | | | *Trichoderma* | | | |
| *Trichoderma* | 1 | RD1 | - | - | 3—4 | 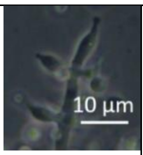 | 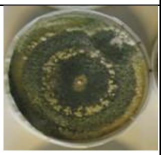 |
| | | | | *Chaetomium* | | | |

| | | | | | | | |
|---|---|---|---|---|---|---|---|
| *Chaetomium* | 2 | SF1 | - | - | 9—10 | 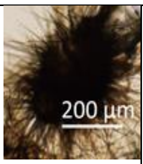 | 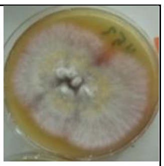 |
| | | | *Paecilomyces* | | | | |
| Paecilomyces | 8 | SF3a<br>SF3b<br>SF6<br>SF7 | (+) | + | 5—6 | 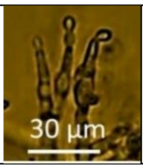 | 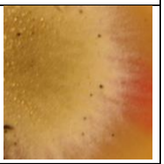 |
| | | | *Penicillium* | | | | |
| Penicillium | 5 | SF5<br>SF1<br>RD1<br>RD2 | - | - | 3—4 | 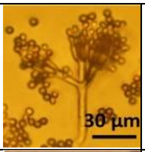 | 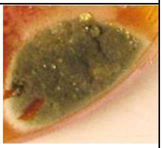 |
| Penicillium | 10 | RD1<br>RD2 | - | - | 2—3 | 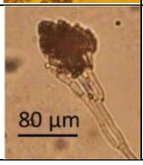 | 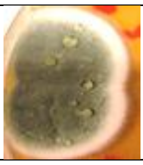 |
| | | | Yeast | | | | |
| Yeast | 4 | SF2b | + | + | | 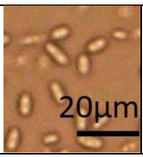 | 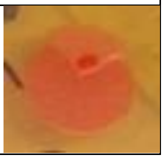 |

* += Growth on TSA and MEA similar, i.e. colonies of same size on TSA and MEA, (+) growth weeker on TSA than MEA. – No visible growth on TSA detected.
